# Why avoid naming diseases after animals? The case of “*Molluscum contagiosum*”

**DOI:** 10.1186/s41182-024-00586-4

**Published:** 2024-03-06

**Authors:** Fabrizio M. Machado, Rodrigo B. Salvador

**Affiliations:** 1 Departamento de Biologia Animal, Instituto de Biologia, Universidade Estadual de Campinas, Campinas, SP CEP 13083-970 Brazil; 2 The Arctic University Museum of Norway, UiT-The Arctic University of Norway, Lars Thørings Veg 10, 9006 Tromsø, Norway

**Keywords:** Water warts, Verrugas de água, Poxviridae, Mollusca, Animal welfare

## Abstract

For over 200 years, the name molluscum contagiosum—a dermatological disease—has unfairly associated molluscs (the second largest group of animals on the planet) with this highly contagious infectious disease. Herein, arguments are presented demonstrating the serious problem of continuing to use this name, including animal welfare concerns. Thus, to minimize any unnecessary impacts on the biodiversity and conservation of molluscs, we follow WHO best practices in naming diseases to suggest the use of the new term ‘wpox’ or ‘water warts’ as a synonym for molluscum contagiosum.

First noticed in 1796 by British naturalist and physician Edward Jenner, the father of vaccination, hydatids (from Greek *hudatis* = ‘watery vesicle/cyst’) appeared on his son Edward’s face and neck causing itching and inflammation around these small vesicles and, after a while, spread to his wife’s lips [1]. Jenner, however, did not publish his observation. Eighteen years later, British physician Thomas Bateman (1778–1821) coined the name *molluscum* (from Latin *molis* = ‘soft’), probably due to the lesions/tubercles from which he managed to express a ‘soft’ milky fluid [2, 3].

Currently, it is known that *molluscum contagiosum* (or MC) is a self-limited cutaneous viral infection that affects mainly children, sexually active adults, and immunosuppressed individuals [4, 5]. It is one of the 50 most prevalent diseases worldwide, representing around 1% of all diagnosed skin disorders and thus being a public health issue [6]. The infection is caused by a double-stranded DNA poxvirus known as *molluscum contagiosum virus* (MCV) and belonging to the *Molluscipoxvirus* genus, family Poxviridae [4, 7].

The term *molluscum* on the name of this disease has, for over two centuries, given the false idea that molluscs (members of the animal phylum Mollusca) are directly or indirectly associated with it. This generates not only a stigmatization of this extremely biodiverse animal phylum, but also a negative and erroneous association between molluscs and this highly contagious infectious dermatologic disease. Furthermore, the name *molluscum contagiosum* fails greatly to present the true etiological agent (a virus), probably making dermatologists and paediatricians uncomfortable and confused explaining this condition to their patients. Traditionally, only zoonoses were named after animals or properly styled as zoonymic (e.g., mad cow disease, swine flu, avian flu, cat scratch fever, monkey pox, etc.) [8], which also reinforces the non-standard use of the term *molluscum contagiosum*. Not even the whitish substance/cheesy material (termed ‘molluscum body’ or Henderson-Patterson bodies) within the tubercles, vesicles, pustules, bumps or waxy papules (= skin lesions, known as ‘mollusca’) observed in the patient’s skin is always “soft” (= *molis*) [9]. In fact, it sometimes has a liquid or even fluid consistency. So why was the name *molluscum contagiosum* accepted and consolidated in the scientific literature?

The disease was given other names in the literature along the decades after its first description, such as ‘molluscum verrucosum’, ‘molluscum epitheliale’, ‘molluscum tumors’, ‘acne varioliform’, and ‘epithelioma contagiosum’ [10]. It was only in 1899, that a publication in JAMA (The Journal of the American Medical Association), after acceptance by most English-speaking dermatologists, ratified the use of the mistaken and unfortunate name *molluscum contagiosum* [10]. According to the latter author, the decision was taken after the study of Dr Henry W. Stelwagon, an American dermatologist who proved the contagiousness of the disease [11]. To this day, ramifications of this name (e.g., ‘molluscum dermatitis’, ‘eczema molluscorum’) are being created (e.g., [12], perpetuating the mistake of two centuries ago.

It is also necessary to understand who the animals stigmatized by this name are and why we (scientists, zoologists and physicians) should propose a synonym for the term *molluscum contagiosum*. The Phylum Mollusca (e.g., octopuses, squids, snails, slugs, clams, oysters, mussels) was proposed by Carl Linnaeus in 1758, currently being the second (after arthropods) most diverse group of animals on the planet, with about 85,000 known living species [13]. Molluscs are essential for the healthy functioning of ecosystems on land and in the sea and are, in several cases, considered to be ecosystem engineers [14, 15].

The problem with the disease’s name is reinforced in some Romance languages like Spanish and Portuguese. The translation of the name *molluscum contagiosum* from scientific Latin to vernacular Spanish and Portuguese results in “molusco contagioso”, which is not only a homonym with molluscan animals, but also imply, by the word order, that the animal is the source of contagion. This further strengthens the false relationship between etiologic agent and disease.

Notably, in some countries like the USA and The Netherlands, the disease is known vernacularly as ‘water warts’ (‘waterwratten’ in Dutch)*,* a concept similar to the water vesicles (= hydatids) noted by Edward Jenner in 1796.

Thus, the name *molluscum contagiosum* continues to cause prejudice and disinformation, mainly amidst the general public, which is more than enough reason to phase it out from the literature and common usage. This proposal has reasonable precedence, as a very similar case has recently received wide attention. When news started spreading about monkeypox in 2022, the fear of the disease drove people in Brazil to persecute, poison, attack, and kill monkeys [16]. That forced the WHO to make a pronouncement to state that the animals were not contagious and should not be killed [17]. The name of the disease in that case, was due to the virus having been discovered in captive monkeys in 1958. Later, the WHO proposed the use of term “mpox” as a synonym for and substitute of “monkeypox”, due to the use of the term as racist and stigmatizing language in some communities [18]. That decision has been well accepted by the community [19] and has also worked in favor of the animals, particularly when considering that primates are the most endangered mammal group in Brazil [20].

According to the WHO’s guide of best practices in naming diseases from 2015, disease names should avoid unnecessary negative impact on, among many other things, animal welfare. Thus, to minimize any unnecessary impacts on the biodiversity and conservation of molluscs, we propose changing the disease’s name in the WHO International Classification of Diseases (ICD). We suggest the use of ‘wpox’ or ‘water warts’ (the latter a name already widely used in some countries) as synonyms of “*molluscum contagiosum*” (ICD-10: B8.01). Such a change would only affect the vernacular name of the disease and not the scientific nomenclature of the virus. Our suggestion takes into consideration current usage, scientific accuracy and appropriateness, as well as linguistic concerns (e.g., pronounceability, translatability), thereby reducing the potential for confusion and negative impacts. Regarding translatability, in particular to the languages singled out above where the nomenclatural issues was more problematic (i.e., Spanish and Portuguese), the new name could be easily rendered as ‘verrugas de agua’ in the former and ‘verrugas d’água’ in the latter.

Considering the good uptake by community of the mpox change, we are optimistic that our proposal can be adopted without detrimental effects to research or medicine and for the benefit of wildlife.

## Data Availability

Not applicable.
